# Clinical presentation and outcomes of diabetic ketoacidosis in pediatric and adolescent patients at a tertiary hospital in Jordan

**DOI:** 10.3389/fped.2025.1635037

**Published:** 2025-07-22

**Authors:** Hanan Al Thiabat, Sara Elbanna, Mohammad Quadier, Mohmmad Dayyeh, Bashar Abunnadi, Hadeel Obeidat, Ala'a Abu Shakhdam, Hala Elfarraj, Miral A. Almomani, Faisal Abu-Ekteish

**Affiliations:** ^1^ Department of Pediatrics and Neonatology, Faculty of Medicine, King Abdullah University Hospital, Jordan University of Science and Technology, Irbid, Jordan; ^2^ Faculty of Medicine, Jordan University of Science and Technology, Irbid, Jordan

**Keywords:** diabetic ketoacidosis, type 1 diabetes mellitus, outcome, clinical presentation, cerebral edema

## Abstract

**Introduction:**

Diabetic ketoacidosis (DKA) remains a serious and potentially life-threatening complication of diabetes in children and adolescents, particularly in resource-limited settings, with overall mortality of 3.4%–13.4% in developing countries. Understanding the clinical presentation, treatment outcomes, and complication rates is essential to improving care and reducing morbidity.

**Methods:**

A retrospective analysis was conducted on pediatric patients aged 2–16 years admitted with DKA to King Abdulla University Hospital (KAUH) between 2017 and 2023. Data were collected on age, diagnosis status, DKA severity, and clinical outcomes.

**Results:**

DKA was most common between 6 and 12 years old (67.8%) with mean age of 9 (IQR = 5). More than two thirds of children were newly diagnosed upon admission (72.4%) while the remainder were known cases of T1DM, 41.1% of patients had severe DKA while 35.7% had moderate DKA. No mortality was reported during the study period.

**Conclusion:**

DKA remains a frequent presentation among newly diagnosed pediatric diabetes cases in this setting, with a high proportion of moderate to severe presentations. Despite the severity, no deaths were reported, highlighting the effectiveness of management protocols at KAUH. Continued efforts are needed to improve early diagnosis and reduce complication rates.

## 1 Introduction

DKA (diabetic ketoacidosis) is a sequel resulting from a severe deficiency of insulin. It is a common pediatric endocrine emergency and one of the most common chronic endocrine disorders that affects children and adolescents worldwide (1). Spreading the knowledge about early clinical symptoms such as polyuria and polydipsia can prevent serious complications and fatal outcomes (2).

According to The International Society for Pediatric and Adolescent Diabetes (ISPAD) 2022, The biochemical criteria for the diagnosis of DKA are Hyperglycemia [blood glucose >11 mmol/L (≈200 mg/dl)], Venous pH <7.3 or serum bicarbonate <18 mmol/L and Ketonemia (blood ß-hydroxybuyrate ≥3 mmol/L) or moderate to large ketonuria (3). DKA severity was categorized based on the degree of acidosis at admission: mild (pH < 7.3 or bicarbonate <18 mmol/L), moderate (pH < 7.2 or bicarbonate <10 mmol/L), or severe (pH < 7.1 or bicarbonate <5 mmol/L) (3).

In 2016, it was estimated that approximately 65,000 children aged under 15 develop Type 1 diabetes mellitus (T1DM) each year worldwide, and 13%–80% of these children present with DKA at the time of diagnosis; this varies according to the region (4). With the rising incidence of T1DM, several studies have been conducted to determine the prevalence and different associations (5).

Starting in Jordan, studies conducted at Jordan University Hospital revealed a prevalence of 31.7% DKA as the first presentation of T1DM (6). DKA prevalence in the United States increased from 35.3% to 40.6%; the increase in DKA was 2% annually at the diagnosis of T1DM (7). Across Europe, Australia, New Zealand, and the United States, the overall prevalence of DKA was 29.9% (7). Whereas in North India, 48.2% of patients admitted with DKA were newly diagnosed in 2017 (8).

Most DKA patients fully recover as venous pH > 7.3, and serum bicarbonate ≥18 mmol/L, in addition, clinical resolution of symptoms without major complications if proper management is applied, considering the severity of DKA at presentation and other factors (9). Variable complications have been introduced and studied, and it was shown that cerebral edema is one serious complication leading to higher motility levels, with an increasing incidence in younger ages (10). Other complications increasing morbidity and mortality in DKA include hypoglycemia, infections, intracranial hemorrhage or thrombosis, cardiac arrhythmias caused by electrolyte abnormalities, pancreatitis, and renal failure (11).

There is limited published data on the incidence, outcomes, and complications of DKA in North Jordan, so this study was conducted to review the DKA outcomes and complications in children and adolescents at a tertiary care hospital in North Jordan since early recognition is associated with a decreased incidence of complications.

## 2 Materials and methods

This is a retrospective analysis of diabetic ketoacidosis (DKA) outcomes and complications in children and adolescents at King Abdullah University hospital (KAUH), the largest medical structure in the north of Jordan and It is also the teaching hospital affiliated with Jordan University of Science and Technology (JUST). All patients between 2 and 16 years of age diagnosed with DKA from 2017 until 2023 were included. The study involved a list of data collected from the hospital's database. The data was collected by reviewing the clinical records of the patients. DKA is defined as hyperglycemia (random blood sugar >11 mmol/L or approximately 200 mg/dl), venous blood pH < 7.3, or bicarbonate <18 mmol/L, along with ketonemia or ketonuria in urine samples. Patients with other comorbidities or missing data were excluded (Two patients were excluded from the initial cohort of 89. One patient died from complications associated with Wolcott-Rallison syndrome before the age of one, and the other was transferred to another facility shortly after diagnosis).

We recorded clinical presentation, degree of dehydration, gender, and age of the patient at admission, and the severity of DKA, which was classified according to the degree of acidosis and bicarbonate levels, as outlined in the 2022 ISPAD guidelines (3). Mild DKA was defined by a venous pH of less than 7.3 or a serum bicarbonate level below 18 mmol/L; moderate DKA by a pH less than 7.2 or bicarbonate below 10 mmol/L; and severe DKA by a pH less than 7.1 or bicarbonate under 5 mmol/L (3). In addition to biochemical tests such as serum blood glucose, sodium, potassium, phosphate and bicarbonate level. Then we collected other variables during admission, such as number of days spent in the intensive care unit (ICU), follow-up values of sodium, potassium, and phosphorus, renal function tests, and any other complications including cerebral edema or death. These variables provided a solid basis for our analysis, allowing for thorough exploration and interpretation of the study's findings.

The study was approved by the institutional review board (IRB) at Jordan University of Science and Technology. The IRB number is 2023/87 (20230156). Data was entered into Excel and analyzed on IBM SPSS statistics version 28.0 and Jamovi 2.3.28.0. In the descriptive analysis, the mean and percentages were computed. Associations were evaluated using the chi-square test. A *p*-value of <0.05 was considered statistically significant. All ethical considerations were ensured throughout the study.

## 3 Results

This cross-sectional has documented the characteristics of 87 DKA episodes over a period of 7 years admitted at KAUH. The population which consisted of children aged 2–16 years was mostly females (F = 69%, M = 31%) and was most common between 6 and 12 years old (67.8%) with mean age of 9 ((The interquartile range IQR = 5). More than two thirds of children were newly diagnosed upon admission (73.6%) while the remainder were known cases of T1DM (26.4%). The following text will describe the presentation in greater detail.

### 3.1 Baseline characteristics of children admitted with DKA

The study has explored the common presenting symptoms of the patients and showed that most children complained of vomiting, abdominal pain, polyuria, polyphagia, and polydipsia (59%, 53%, 35%, 30% and 30%, respectively) upon admission. Other presentations included fever, general weakness, deep rapid breathing, decreased level of consciousness and lethargy as shown in Table 1.

**Table 1:** Clinical presentation of DKA patients on admission.

Presentation	Frequency *N* (%)
Vomiting	51 (59%)
Abdominal pain	46 (53%)
Polyuria	30 (35%)
Polyphagia	26 (30%)
Polydipsia	26 (30%)
Diarrhea	4 (4.7%)
Fever	4 (4.7%)
General weakness	3 (3.5%)
Deep rapid breathing	2 (2.3%)
Decreased LOC	1 (1.2%)
Lethargy	1 (1.2%)

More than two thirds of patients were newly diagnosed (73.6%) while the remainder were known cases of T1DM. The investigations of admitted children showed different blood glucose levels upon admission 42.5% of which were between 350 and 500 (<350 = 24.1%, >500 = 33.4%). The majority of patients (74.1%) presented with moderate dehydration. At admission, hypokalemia was identified in 5 patients (5.7%), hyponatremia in 55 patients (63.2%), and acute kidney injury in 62 patients (71%) as shown in Tables 2, 3.

### 3.2 Association between severity of DKA & other variables at presentation

We reported the number of patients who had mild, moderate, and severe DKA were 26 (30%), 30 (34%) and 31 (36%), respectively. We found a significant association between fever and severity with a *p*-value of 0.034 (Table 3). Hitherto, we found no significant association between the severity of DKA and age, gender, days at ICU, acute kidney injury and lab investigations at presentation (blood glucose, potassium, and sodium levels (Table 3).

**Table 2:** Baseline characteristics of children admitted with DKA.

Characteristics	Frequency
Gender	
Male	31%
Female	69%
Dehydration	
Mild	24.7%
Moderate	74.1%
Severe	1.2%
Na level at presentation (mmol/L)	
<125	1.1%
125–135	62.1%
135–145	35.6%
>145	1.1%
K level at presentation (mmol/L)	
<3.5	5.7%
3.5–5.1	74.7%
>5.1	19.6%
Blood glucose (mg/dl)	
<350	24.1%
350–500	42.5%
>500	33.4%
Known case of DM	
Yes	26.4%
No	73.6%
Age at diagnosis (years)	
<6	23%
6–12	67.8%
>12	9.2%

**Table 3:** Association between severity of DKA and other variables.

Variables\severity	Mild	Moderate	Severe	*P*-value
AGE (years)				
<6	4	5	11	
6–12	20	22	17	0.331
>12	2	3	3	
Gender				
Male	11	8	8	0.332
Female	15	22	23	
Days in ICU				
1–2	16	14	13	
3–4	8	14	16	0.535
5–6	2	1	2	
7–8	0	1	0	
Fever at presentation	1	1	2	0.034
Na level at presentation (mmol/L)				
<125	0	0	1	
125–135	18	18	18	0.641
135–145	8	12	11	
>145	0	0	1	
K level at presentation (mmol/L)				
<3.5	0	1	4	
3.5–5.1	23	24	18	0.064
>5.1	3	5	9	
Blood glucose (mg/dl)				
<350	5	6	10	
350–500	11	15	11	0.733
>500	9	10	10	
Acute kidney injury				
not injured	7	12	6	0.199
injured	19	18	25	

### 3.3 Association of DKA complications and management with other variables

During management of DKA 21 patients (24%) developed hypokalemia, while 39 (45%) developed hypophosphatemia (Table 4). A statistically significant association was observed between the development of hypokalemia and the severity of DKA (*p* = 0.036), indicating that more severe cases were more likely to develop potassium disturbances during treatment.

**Table 4:** Association between hypokalemia and hypophosphatemia during treatment and other variables.

Variables\complications	Hypokalemia (total 21)	Hypophosphatemia (total 39)
AGE		
<6	6	11
6–12	15	26
>12	0	2
*P*-value	0.275	0.572
Severity		
Mild	1	8
Moderate	10	15
Severe	10	16
*P*-value	0.036	0.075
Days in ICU		
1–2	8	19
3–4	10	16
5–6	2	3
7–8	1	1
*P*-value	0.552	0.511
Degree of dehydration		
Mild	2	6
Moderate	19	33
Severe	0	0
*P*-value	0.426	0.274
DKA at diagnosis		
Known case	4	9
Newly diagnosed	17	30
*P*-value	0.667	0.896
HBA1C		
<6.4	2	3
6.4–8	0	0
8–10	4	4
10–12	7	14
>12	8	18
*P*-value	0.64	0.067

## 4 Discussion

In this retrospective analysis, 87 episodes of DKA according to inclusive criteria were evaluated to describe the characteristics, outcomes, severity, and complications of DKA in North Jordan. Due to the small sample size, the accuracy may be affected, but the significance of the values couldn't be denied, especially with the increasing incidence of T1DM.

DKA as initial presentation carried high morbidity and mortality (8) delayed diagnosis could be to its nonspecific signs and symptoms, which overlap with other illnesses, such symptoms include abdominal pain, vomiting and rapid breathing. And other factors especially in newly diagnosed patients due to lack of awareness of the signs and symptoms among the general population and primary care physician (8).

In the present study, the population which consisted of children aged 2–16 years was mostly females (F = 69%, M = 31%) which was also significant in other studies such as study done in Germany (12), and was most common between 6 and 12 years old (67.8%) with mean age of 9 years old (IQR = 5). More than two thirds of children were newly diagnosed upon admission (73.6%) while the remainder were known cases of T1DM (26.4%).

The most common symptoms at presentation was vomiting (59%), and the other symptoms such as abdominal pain, polyuria, polyphagia, and polydipsia (53%, 35%, 30% and 30%, respectively) and very rare they presented as general weakness (3%), rapid breathing (2%), lethargy (1%) and decreased level of consciousness (1%), on physical exam they mostly presented with moderate signs of dehydration (74.1%).

Severe DKA was in 36% of the cases, whereas moderate cases were in 35%. In the study held in Jordan University Hospital, severe cases were around 28% and moderate cases were 33% (6). This difference in the percentage of severe DKA could be explained by the fact that Jordan University Hospital serves patients in Jordan's capital, where the education level and awareness are better.

The incidence of DKA recurrence was 27%, it was precipitated by acute illness in 38% and 41% due to missed insulin dose.

### 4.1 Complications of DKA and its management

#### 4.1.1 Mortality

The mortality rate from DKA was reported to be 0.15%–0.31% in developed countries (13), but it is higher in developing countries (3.4%–13.4%) (13). No mortality was recorded in this study, which may be attributed to the relatively small sample size. Moreover, the study was conducted at the largest medical facility in northern Jordan, which also serves as a teaching hospital. The availability of experienced medical staff, continuous supervision, and adherence to standardized treatment protocols potentially contribute to effective and timely management of DKA cases.

#### 4.1.2 Cerebral edema

Cerebral edema is still the most serious complication of DKA in children (14), incidence of diabetic ketoacidosis (DKA) occurs in 25%–40% of children with newly diagnosed type 1 diabetes mellitus (14) while the incidence of overt cerebral injury is 0.5%–0.9% (3).

The pathophysiology is not clearly known; some suppose it is added to cerebral hypoperfusion correlating to the severity and dehydration status (2); others suggest that it comes with a hyperinflammatory status where multiple cytokines have been detected with elevated levels at the DKA diagnosis, the most prominent being interleukin 10 (IL-10) (15).

Cerebral injury is more commonly observed in younger children (16), those experiencing their first episode of diabetes, and those with a longer duration of symptoms before presentation. These associations may reflect a higher likelihood of severe DKA in these groups. Epidemiological studies have identified several biochemical risk factors present at diagnosis, including: higher degrees of CO2, elevated serum urea nitrogen levels, and the use of bicarbonate to correct acidosis has been linked to an increased risk of cerebral injury (3).

Cerebral edema was identified in one patient (1.1%) in our cohort. The case involved a 3-year-old female with a known history of type 1 diabetes mellitus who presented in severe diabetic ketoacidosis (pH 6.9, serum bicarbonate 3 mmol/L). At presentation, she was febrile and diagnosed with a concurrent urinary tract infection. On the second day of hospitalization, the patient developed a decreased level of consciousness and was managed with hypertonic saline for suspected cerebral edema. This finding is consistent with previously reported data, where the incidence of cerebral edema in individuals under 16 years of age with DKA in Canada is approximately 0.51%, with an associated mortality rate of 23% (17).

#### 4.1.3 Potassium disturbance

Hyperkalemia at presentation in DKA is typically attributed to a shift of potassium from the intracellular to the extracellular space, driven by acidosis, insulin deficiency, and increased plasma osmolality—factors that contribute to what is often considered pseudo-hyperkalemia (18). In our cohort, hyperkalemia was identified in 19.6% of patients upon admission. Conversely, hypokalemia was present in 5.7% of patients at admission. Our findings are consistent with those reported in adult populations; for example, a study from Los Angeles County Hospital, University of Southern California, documented hypokalemia in 5.6% of adult DKA patients at presentation (19). Similarly, a study of children with DKA in India reported an initial hypokalemia rate of 14.5% (20). Although less frequent initially, early recognition and management of hypokalemia are essential, as it can become life-threatening, especially following the initiation of insulin therapy and correction of acidosis. In our study, 24% of patients developed hypokalemia during treatment, with a statistically significant association found between hypokalemia and the severity of DKA (*p* = 0.036). This indicates that patients with more severe acidosis are at greater risk for treatment-related potassium shifts, emphasizing the need for vigilant electrolyte monitoring and timely potassium replacement, particularly in severe cases. These shifts occur primarily due to insulin-driven intracellular redistribution of potassium following the resolution of acidosis (18).

#### 4.1.4 Hypophosphatemia

Hypophosphatemia in patients with DKA occurs secondary to urinary loss with osmotic diuresis and decrease renal phosphate reabsorption. During treatment, insulin administration and correction of metabolic acidosis promote a shift of phosphate from the extracellular to the intracellular compartment, further exacerbating hypophosphatemia (21). In our study, 45% of patients developed hypophosphatemia during DKA treatment. Although this prevalence is lower than the 77% reported in a Dutch–Australian cohort, both studies underscore the common occurrence of phosphate disturbances during early DKA management in pediatric populations (22).

#### 4.1.5 Acute kidney injury

Another consequence observed in our study was the existence of acute kidney injury (AKI), which had an incidence of 71% at diagnosis (*p* value = 0.19) as shown in Table 3 and resolved in 100% of the patients with fluid correction. This was consistent with Canadian research that showed 64.2 percent of DKA patients had AKI, and it suggested an increased risk of developing severe AKI in the presence of severe acidosis and severe dehydration (23).

### 4.2 Limitation

One of the main limitations of this study is the relatively small number of cases included in the analysis. The limited sample size may affect the generalizability of the findings and reduce the statistical power to detect significant associations or trends.

## 5 Conclusion

This study reveals a significant incidence of severe diabetic ketoacidosis (DKA) at presentation in pediatric patients diagnosed with type 1 diabetes at King Abdullah University Hospital (KAUH), indicating an ongoing necessity for enhanced public and primary care awareness. Despite the severity of presentation, the absence of mortality is a notable outcome and may reflect the benefits of centralized care, timely intervention, and the presence of experienced clinical teams in a tertiary, teaching hospital setting. These findings emphasize the importance of early recognition, prompt referral, and the application of standardized treatment protocols. Strengthening community-level diabetes education, particularly among families and primary care providers, could reduce the frequency of severe presentations.

## References

[1] Lone SW Siddiqui EU Muhammed F Atta I Ibrahim MN Raza J. Frequency, clinical characteristics and outcome of diabetic ketoacidosis in children with type-1 diabetes at a tertiary care hospital. J Pak Med Assoc. (2010) 60(9):725–9. 21381577

[2] Choi AY Park E. The impact of pediatric intensivists on the management of pediatric diabetic ketoacidosis in pediatric intensive care units: BMC pediatrics. BMC Pediatr. (2023) 23(1):562. 10.1186/s12887-023-04398-z 37957591 PMC10644449

[3] Glaser N Fritsch M Priyambada L Rewers A Cherubini V Estrada S ISPAD clinical practice consensus guidelines 2022: diabetic ketoacidosis and hyperglycemic hyperosmolar state. Pediatr Diabetes. (2022) 23(7):835–56. 10.1111/pedi.13406 36250645

[4] Shaltout AA Channanath AM Thanaraj TA Omar D Abdulrasoul M Zanaty N Ketoacidosis at first presentation of type 1 diabetes mellitus among children: a study from Kuwait. Sci Rep. (2016) 6(1):27519. 10.1038/srep27519 27328757 PMC4916451

[5] DIAMOND Project Group. Incidence and trends of childhood type 1 diabetes worldwide 1990–1999. Diabet Med. (2006) 23(8):857–66. 10.1111/j.1464-5491.2006.01925.x 16911623

[6] Odeh R Gharaibeh L Daher A Albaramki J Ashour B Barakat FA Frequency, clinical characteristics and predictors of ketoacidosis at diagnosis of type one diabetes mellitus in children and adolescents from Jordan. J Clin Res Pediatr Endocrinol. (2023) 15(1):46–54. 10.4274/jcrpe.galenos.2022.2022-5-8 36264035 PMC9976172

[7] Virdi N Poon Y Abaniel R Bergenstal RM. Prevalence, cost, and burden of diabetic ketoacidosis. Diab Technol Ther. (2023) 25(S3):S75–84. 10.1089/dia.2023.0149 37306442

[8] Bhardwaj P Yadav V Sharma M. Clinical profile and outcome of the children with diabetic ketoacidosis (DKA) in Hilly Himalayan state of North India. Int J Res Med Sci. (2017) 5(12):5402. 10.18203/2320-6012.ijrms20175463

[9] Seo Y-J Kum CD Rho JG Shim YS Lee HS Hwang JS. Comparison of the clinical characteristics and outcomes of pediatric patients with and without diabetic ketoacidosis at the time of type 1 diabetes diagnosis. Ann Pediatr Endocrinol Metab. (2022) 27(2):126–33. 10.6065/apem.2142174.087 35073669 PMC9260367

[10] Lizzo JM Goyal A Gupta V. Adult Diabetic Ketoacidosis. Treasure Island, FL: StatPearls Publishing (2023). Available online at: https://www.ncbi.nlm.nih.gov/books/NBK560723/32809558

[11] Abbas Q Arbab S Haque AU Humayun KN. Spectrum of complications of severe DKA in children in pediatric intensive care unit. Pak J Med Sci. (2018) 34(1):106–9. 10.12669/pjms.341.13875 29643888 PMC5856992

[12] Neu A Willasch A Ehehalt S Hub R Ranke MB, DIARY Group Baden-Wuerttemberg. Ketoacidosis at onset of type 1 diabetes mellitus in children–frequency and clinical presentation. Pediatr Diabetes. (2003) 4(2):77–81. 10.1034/j.1399-5448.2003.00007.x 14655263

[13] Varadarajan P Poovazhagi V. Risk factors for mortality in children with diabetic keto acidosis from developing countries. World J Diabetes. (2014) 5(6):932–8. 10.4239/wjd.v5.i6.932 25512799 PMC4265883

[14] Shastry RM Bhatia V. Cerebral edema in diabetic ketoacidosis. Indian Pediatr. (2006) 43(8):701–8. 16951433

[15] Hoffman WH Burek CL Waller JL Fisher LE Khichi M Mellick LB. Cytokine response to diabetic ketoacidosis and its treatment. Clin Immunol. (2003) 108(3):175–81. 10.1016/s1521-6616(03)00144-x 14499240

[16] Rosenbloom A. Intracerebral crises during treatment of diabetic ketoacidosis. Diabetes Care. (1990) 13:22–33. 10.2337/diacare.13.1.22 2105195

[17] Lawrence SE Cummings EA Gaboury I Daneman D. Population-based study of incidence and risk factors for cerebral edema in pediatric diabetic ketoacidosis. J Pediatr. (2005) 146(5):688–92. 10.1016/j.jpeds.2004.12.041 15870676

[18] Kaveriappa PD Sumithra S Upadhya PYN Bhat NK. Severe hyperkalemia in a child with diabetic ketoacidosis: a case report J Pediatr Crit Care. (2023) 10(6):280–2. 10.4103/jpcc.jpcc_68_23

[19] Arora S Cheng D Wyler B Menchine M. Prevalence of hypokalemia in ED patients with diabetic ketoacidosis. Am J Emerg Med. (2012) 30(3):481–4. 10.1016/j.ajem.2011.01.002 21316179

[20] Kanwal SK Bando A Kumar V. Clinical profile of diabetic ketoacidosis in Indian children. Indian J Pediatr. (2012) 79:901–4. 10.1007/s12098-011-0634-3 22207489

[21] Levine BS Ho K Kraut JA Coburn JW Kurokawa K. Effect of metabolic acidosis on phosphate transport by the renal brush-border membrane. Biochim Biophys Acta. (1983) 727(1):7–12. 10.1016/0005-2736(83)90362-0 6824655

[22] Hasan RA Hesen JZ Millican N Pederson JM Agus MSD. Serum phosphorus and hypophosphatemia during therapy of diabetic ketoacidosis in children: single-center, retrospective cohort 2016–2022. Pediatr Crit Care Med. (2025) 26(1):e77–85. 10.1097/PCC.0000000000003649 39785552 PMC11706349

[23] Hursh BE Ronsley R Islam N Mammen C Panagiotopoulos C. Acute kidney injury in children with type 1 diabetes hospitalized for diabetic ketoacidosis. JAMA Pediatr. (2017) 171(5):e170020. 10.1001/jamapediatrics.2017.0020 28288246

